# Chitin Oligosaccharide and Chitosan Oligosaccharide: Two Similar but Different Plant Elicitors

**DOI:** 10.3389/fpls.2016.00522

**Published:** 2016-04-22

**Authors:** Heng Yin, Yuguang Du, Zhongmin Dong

**Affiliations:** ^1^Department of Biotechnology, Dalian Institute of Chemical Physics, Chinese Academy of SciencesDalian, China; ^2^National Key Laboratory of Biochemical Engineering, Institute of Process Engineering, Chinese Academy of SciencesBeijing, China; ^3^Department of Biology, Saint Mary's UniversityHalifax, NS, Canada

**Keywords:** chitin oligosaccharides, chitooligosaccharides, chitosan oligosaccharides, plant immunity, plant resistance

## Introduction

Natural resources have been traditionally used in agriculture by humans. For example, crab and shrimp shell powder has been applied to control crop disease and improve soil fertility (Ha and Huang, 2007). On the other hand, chitin is an important structural component in fungal cell walls and can be degraded by plant chitinases to eradicate fungal infection (Grover, 2012). Plant cells can recognize chitin and chitin-derived molecules to elicit immune response. Since the 1980s, chitin and its deacetylation product chitosan have been used for crop farming as biopesticides, biofertilizers, seed coating formulation, and agricultural film (El Hadrami et al., 2010; Hadwiger, 2013; Trouvelot et al., 2014).

In order to overcome the poor solubility obstacle to chitin and chitosan application, soluble chitin oligosaccharides (CTOS) and chitosan oligosaccharides (CSOS) are prepared from these polysaccharides. The effects of CTOS and CSOS on crop disease control were validated by several researches (Yin et al., 2010). Several biopesticides or biofertilizers have been developed based on these two oligosaccharides. However, the quantity and quality of the research on CTOS is much better than that on CSOS. Some papers even claimed that CSOS had no effect on plant disease control (Vander et al., 1998). However, based on the previous works (Cabrera et al., 2006; Maksimov et al., 2011; Guo et al., 2012), just like CTOS, CSOS are potent pathogen-associated molecular patterns (PAMP).

## Chemical and physical properties

Chitin is the second most abundant biomass in the world and can be found in fungal cell walls and the exoskeletons of arthropods (Rinaudo, 2006). Compared to chitin, chitosan is rare in nature and found only in the fungi that have deacetylase enzymes. CTOS and CSOS are the degradation products of chitin and chitosan. CTOS and CSOS are builtup with N-acetylglucosamine or glucosamine with a degree of polymerization (DP) from 2 to 10. The only difference is the acetyl group on the C2 of the sugar ring in CTOS. The degree of acetylation (DA) is closely linked with the polysaccharides material, the chemical, or enzyme degradation methods, as well as modification of enzymes such as chitin deacetylase (Tsigos et al., 2000). The acetyl group is important to CTOS function (Maksimov et al., 2011). For example, acetyl group is important to CTOS binding with its receptor (Liu et al., 2012; Hayafune et al., 2014). On the other hand, CSOS, with only an amino group on C2, are cationic oligosaccharides, which are attracted to negatively charged materials, such as plant cell membrane. This character makes the function and mechanism of CSOS more complicated.

Another issue in this field is the ability to define and differentiate CTOS and CSOS. Theoretically, CTOS should have a 100% DA and CSOS should have no acetylation. However, lots of oligosaccharides, called chitooligosaccharides (COS), contain both N-acetylglucosamine and glucosamine because of the varied preparation methods (Yin et al., 2009; Jung and Park, 2014). Previous reports showed that COS may have different functions and functional mechanisms with a primary factor of DA difference (Falcon et al., 2008). So, we strongly recommend that scientists and companies provide the detailed DA data when they discuss CTOS or CSOS, which should only be described as with the 100 and 0% DA oligosaccharides.

CTOS and CSOS, the degradation products of chitin and chitosan, have different physical characteristics from the polysaccharides, which are important for their functions (Kim and Rajapakse, 2005; Jung and Park, 2014). Chitin is insoluble in common solvents, whereas chitosan dissolves in aqueous acidic solutions. Low DP CTOS ranging from 2 to 6 can be dissolved in neutral water. However, the CTOS with DP > 6 are not easily dissolved in neutral water, which limits its application. On the other hand, all CSOS have a good solubility in neutral water.

CSOS production is inexpensive and effective using several advanced methods including physical, chemical, and biological techniques. Nowadays, the environmentally friendly enzymatic method is favored as a near perfect process, which can control the DP and DA of the products with low-cost (Jung and Park, 2014). However, CTOS production is still challenging with a small quantity of commercial products using chemical methods. Though many chtinases have been reported, enzymatic production of CTOS is not very successful.

## Signal recognition

It remains unclear about the molecular mechanisms for plants recognizing and transducing the signals of oligosaccharides (Côté and Hahn, 1994; Yin et al., 2010; Trouvelot et al., 2014). Generally, there are five steps for the process: oligosaccharins signal recognized by the receptor on plant cell membrane; signal transfer and magnification; responding-genes activation and responding-proteins accumulation; induction of defense-related secondary metabolites; defense reaction (Yin et al., 2010). The first two steps—the signal recognition and transduction vary greatly among oligosaccharins including CTOS and CSOS.

It is pioneering work in the field of plant immunity for identifying the high-affinity binding protein of CTOS on the surface and microsomal membranes of rice cells (Shibuya et al., 1993). From 1993 to 2002, several other high-affinity binding proteins of CTOS were identified in rice, wheat, barley, soybean, and carrot by affinity labeling and cross-linking of carbohydrate and protein techniques (Shibuya et al., 1996; Kaku et al., 1997; Day et al., 2001; Shibuya and Minami, 2001). A breakthrough was reported in 2006 when Shibuya's lab purified and identified an CTOS-binding protein (CEBiP) from plasma membrane of rice cells. This protein contains the chitin binding lysin motif (LysM) domain, but no intracellular domain, which suggests that it is a component of the CTOS recognition complex (Kaku et al., 2006). Subsequently, another LysM protein, called Chitin Elicitor Receptor Kinase 1 (CERK1) or LysM Receptor-Like Kinase 1 (LysM RLK1) which was cooperated with CEBiP for CTOS recognition, was found soon by Naoto Shibuya's and Gary Stacey's labs respectively (Miya et al., 2007; Wan et al., 2008). CERK1 has the transmembrane and intracellular domains, and several papers suggest that CERK1 is the important component in CTOS recognition (Petutschnig et al., 2010).

In 2012, the crystal structure of AtCERK1 was characterized (Liu et al., 2012). The structure indicates that DP is important to CTOS recognition. The chitin octamer induces the ectodomain of AtCERK1 dimerization, but low DP CTOS can inhibit this effect. The N-acetylglucosamines groups bind with the LysM domain of AtCERK1. The interactions are established mainly through some of the branched groups from one side of N-acetylglucosamines, providing numerous hydrogen bonds with the main chain of AtCERK1 ectodomain. The recognition of the N-acetyl moieties allows AtCERK1 to distinguish CTOS from CSOS and oligoglucan (Liu et al., 2012).

The same binding property has also been found in OsCEBiP binding with CTOS. One chitin oligosaccharide binds two dimerized CEBiPs simultaneously from opposite sides. The dimerization and chitin octamer induces reactive oxygen generation, which is inhibited by a unique chitooligosaccharide (GlcNβ1,4GlcNAc) with acetyl groups on one side of the molecule, suggesting the acetyl groups are essential for binding (Hayafune et al., 2014).

After these breakthroughs on rice and Arabidopsis, lots of CEBiP and CERK1 analogs have been reported from other plants including tomato and maize (Tanaka et al., 2010; Fliegmann et al., 2011; Gust et al., 2012; Zeng et al., 2012; Lee et al., 2014) suggesting that the CTOS recognition is universal in both monocotyledons and dicotyledons. These results rapidly expand the knowledge of CERK1 downstream signal transduction. Several important signal nodes like Rac1, PBL27, LIK1, and RLCK176 have been identified recently (Ao et al., 2014; Le et al., 2014; Shinya et al., 2014). A representative pathway is: OsRacGEF1 phosphorylation dependent with OsCERK1 leads to the activation of the small GTPase OsRac1; OsRac1 activates the MAPK pathway through MKK4 and OsMPK3/6; the activated OsMPK3/6 inspires the downstream transcription factors (Akamatsu et al., 2013). Based on these excellent research, the model of CTOS recognition and signal transduction on arabodiposis or rice is basically clear (Sánchez-Vallet et al., 2014; Shinya et al., 2015). Besides the LysM domain containing-Chitin and CTOS binding protein, plant derived 6-Cys hevein-like peptides could bind with chitin and CTOS (Kini et al., 2015). The biological effect of these hevein-like peptides needs further researches.

However, the research on CSOS recognition is severely lagged behind that of CTOS and reports on the binding proteins of CSOS are very limited. The only reported CSOS binding protein in plants was purified by chitosan affinity chromatography from cultured cells of Rubus (Liénart et al., 1991). Specific binding of CSOS with high affinity to strawberry, tobacco, and rapeseed has been revealed in our lab by using the fluorescent labeling method (Yin and Du, 2009). Concentration and time dependent manner was also observed in these experiments (Guo et al., 2012; Yin et al., 2013). Two CSOS binding proteins have been identified in tobacco and Arabidopsis plasma membrane by affinity chromatography method (Yin et al., 2009). The protein from tobacco is 75 kD, which is similar to the reported chitin oligosaccharides receptor CERK1 with transmembrane and intracellular domains. The structural and functional characteristics of this protein should be further studied to determine if this protein is a receptor. The protein from the Arabidopsis is quite small (12 kD), suggesting that it may not be a receptor.

Another point of view proposes that CSOS has no specific receptors in plants. The evidence is that in former CTOS binding work, CSOS was usually used as an inhibitor and showed no binding ability to CERK1 and CEBiP (Kaku et al., 2006; Miya et al., 2007). The cationic property of CSOS was considered as the primary reason for the binding of CSOS with plant plasma membrane. However, in our experiment, another oligomeric cationic material poly-L-lysine (MW 500–2000 D) cannot inhibit the binding of CSOS to rapeseed membrane, which suggests the binding is not only dependent on the cationic property (Yin et al., 2013). However, it is still uncertain if there are other proteins besides CERK1 that can bind with CSOS.

Besides the signal recognition via a receptor mode, CSOS may also enter the plant nucleus and have the ability to act on chromatin. The chromatin conformational changes can regulate the genes expression directly without the requirement for specific transcription factors (Hadwiger, 2008, 2013).

## Effect and application

In different plant pathosystems, the effects of CTOS, CSOS, and COS with different DAs are distinct and chaotic. The effect is also related to the concentration, DP, application time and methods, and growth period of plants (El Hadrami et al., 2010; Yin et al., 2010; Hadwiger, 2013). Though there are comparison experiments between CTOS and CSOS, we suggest that only the simultaneous experiments in a set of experiments can reflect the real situation. It is a pity that these reports are still rare. Based on above CTOS and CSOS research situation and results, it can only be concluded that both CTOS and CSOS have positive effects on disease control although possibly with different targets.

Interestingly, the applications of CTOS and CSOS are nearly diametrically opposite with their signal research situation. Taking China as an example, there is 55 CSOS-based products from 40 companies with China pesticide registration certificates, but no CTOS products are currently available. This phenomenon is partially due to the mature production techniques of CSOS than those of CTOS.

Taken together, the present situation and problems of CTOS and CSOS research and application can be summarized as following:

1. The large-scale production technology of CSOS is mature and widely used; meanwhile the CTOS production technology is the bottleneck for its application.
2. The research to uncover functional mechanism of CTOS is much more intensive than that of CSOS, especially on the signal recognition and transduction.
3. Although commercial products have been developed and applied to agriculture, the method of application of CTOS and CSOS is not optimized due to the lack of understanding of the related mechanisms.

To solve these problems, the following work should be focused on in the future:

1. Large-scale production technology of CTOS;
2. DA and DP controlled production technology;
3. CSOS signal recognition and transduction;
4. Effect comparison between CTOS and CSOS in the same experiment;
5. Standard application methods of CTOS and CSOS for different plants and their diseases.

## Author contributions

HY, YD, and ZD proposed the idea. HY wrote the paper. YD and ZD revised the paper.

### Conflict of interest statement

The authors declare that the research was conducted in the absence of any commercial or financial relationships that could be construed as a potential conflict of interest.

## References

[B1] Akamatsu A. Wong H. L. Fujiwara M. Okuda J. Nishide K. Uno K.. (2013). An OsCEBiP/OsCERK1-OsRacGEF1-OsRac1 module is an essential early component of chitin-induced rice immunity. Cell Host Microbe 13, 465–476. 10.1016/j.chom.2013.03.007 23601108

[B2] Ao Y. Li Z. Feng D. Xiong F. Liu J. Li J.-F.. (2014). OsCERK1 and OsRLCK176 play important roles in peptidoglycan and chitin signaling in rice innate immunity. Plant J. 80, 1072–1084. 10.1111/tpj.12710 25335639

[B3] Cabrera J. C. Messiaen J. Cambrier P. Van Cutsen P. (2006). Size, acetylation and concentration of chitooligosacharide elicitor determine the switch from defense involving PAL activation to cell death and water peroxide production in Arabidopsis cell suspension. Physiol. Plant. 127, 44–56. 10.1111/j.1399-3054.2006.00677.x

[B4] Côté F. Hahn M. G. (1994). Oligosaccharins – structures and signal-transduction. Plant Mol. Biol. 26, 1379–1411. 10.1007/BF00016481 7858196

[B5] Day R. B. Okada M. Ito Y. Tsukada K. Zaghouani H. Shibuya N.. (2001). Binding site for chitin oligosaccharides in the soybean plasma membrane. Plant Physiol. 126, 1162–1173. 10.1104/pp.126.3.1162 11457966 PMC116472

[B6] El Hadrami A. Adam L. R. El Hadrami I. Daayf F. (2010). Chitosan in plant protection. Mar. Drugs 8, 968–987. 10.3390/md8040968 20479963 PMC2866471

[B7] Falcon A. B. Cabrera J. C. Costales D. Ramirez M. A. Cabrera G. Toledo V. (2008). The effect of size and acetylation degree of chitosan derivatives on tobacco plant protection against *Phytophthora parasitica nicotianae*. World J. Microbiol. Biotechnol. 24, 103–112. 10.1007/s11274-007-9445-0

[B8] Fliegmann J. Uhlenbroich S. Shinya T. Martinez Y. Lefebvre B. Shibuya N.. (2011). Biochemical and phylogenetic analysis of CEBiP-like LysM domain-containing extracellular proteins in higher plants. Plant Physiol. Biochem. 49, 709–720. 10.1016/j.plaphy.2011.04.004 21527207

[B9] Grover A. (2012). Plant chitinases: genetic diversity and physiological roles. Crit. Rev. Plant Sci. 31, 57–73. 10.1080/07352689.2011.616043

[B10] Guo W. Yin H. Ye Z. Zhao X. Yuan J. Du Y. (2012). A comparison study on the interactions of two oligosaccharides with tobacco cells by time-resolved fluorometric method. Carbohydr. Polym. 90, 491–495. 10.1016/j.carbpol.2012.05.070 24751069

[B11] Gust A. A. Willmann R. Desaki Y. Grabherr H. M. Nuernberger T. (2012). Plant LysM proteins: modules mediating symbiosis and immunity. Trends Plant Sci. 17, 495–502. 10.1016/j.tplants.2012.04.003 22578284

[B12] Ha M. T. Huang J. W. (2007). Control of Fusarium wilt of asparagus bean by organic soil amendment and microorganisms. Plant Pathol.Bull. 16, 169–180.

[B13] Hadwiger L. A. (2008). Pea-*Fusarium solani* interactions contributions of a system toward understanding disease resistance. Phytopathology 98, 372–379. 10.1094/phyto-98-4-0372 18944184

[B14] Hadwiger L. A. (2013). Multiple effects of chitosan on plant systems: solid science or hype. Plant Sci. 208, 42–49. 10.1016/j.plantsci.2013.03.007 23683928

[B15] Hayafune M. Berisio R. Marchetti R. Silipo A. Kayama M. Desaki Y.. (2014). Chitin-induced activation of immune signaling by the rice receptor CEBiP relies on a unique sandwich-type dimerization. Proc. Natl. Acad. Sci. U.S.A. 111, E404–E413. 10.1073/pnas.1312099111 24395781 PMC3903257

[B16] Jung W. J. Park R. D. (2014). Bioproduction of Chitooligosaccharides: present and perspectives. Mar. Drugs 12, 5328–5356. 10.3390/md12105328 25353253 PMC4245534

[B17] Kaku H. Nishizawa Y. Ishii-Minami N. Akimoto-Tomiyama C. Dohmae N. Takio K.. (2006). Plant cells recognize chitin fragments for defense signaling through a plasma membrane receptor. Proc. Natl. Acad. Sci. U.S.A. 103, 11086–11091. 10.1073/pnas.0508882103 16829581 PMC1636686

[B18] Kaku H. Shibuya N. Xu P. L. Aryan A. P. Fincher G. B. (1997). N-acetylchitooligosaccharides elicit expression of a single (1->3)-beta-glucanase gene in suspension-cultured cells from barley (*Hordeum vulgare*). Physiol. Plant 100, 111–118. 10.1111/j.1399-3054.1997.tb03460.x

[B19] Kim S. K. Rajapakse N. (2005). Enzymatic production and biological activities of chitosan oligosaccharides (COS): a review. Carbohydr. Polym. 62, 357–368. 10.1016/j.carbpol.2005.08.012

[B20] Kini S. G. Nguyen P. Q. Weissbach S. Mallagaray A. Shin J. Yoon H. S.. (2015). Studies on the chitin binding property of novel cysteine-rich peptides from *Alternanthera sessilis*. Biochemistry 54, 6639–6649. 10.1021/acs.biochem.5b00872 26467613

[B21] Le M. H. Cao Y. Zhang X.-C. Stacey G. (2014). LIK1, A CERK1-interacting kinase, regulates plant immune responses in *Arabidopsis*. PLoS ONE 9:e102245. 10.1371/journal.pone.0102245 25036661 PMC4103824

[B22] Lee W.-S. Rudd J. J. Hammond-Kosack K. E. Kanyuka K. (2014). *Mycosphaerella graminicola* LysM effector-mediated stealth pathogenesis subverts recognition through both CERK1 and CEBiP homologues in wheat. Mol. Plant Microbe Interact. 27, 236–243. 10.1094/mpmi-07-13-0201-r 24073880

[B23] Liénart Y. Gautier C. Domard A. (1991). Isolation from *Rubus* cell-suspension cultures of a lectin specific for glucosamine oligomers. Planta 184, 8–13. 10.1007/BF00208229 24193922

[B24] Liu T. Liu Z. Song C. Hu Y. Han Z. She J.. (2012). Chitin-induced dimerization activates a plant immune receptor. Science 336, 1160–1164. 10.1126/science.1218867 22654057

[B25] Maksimov I. V. Valeev A. Sh. Safin R. F. (2011). Acetylation degree of chitin in the protective response of wheat plants. Biochemistry (Moscow) 76, 1342–1346. 10.1134/S0006297911120078 22150279

[B26] Miya A. Albert P. Shinya T. Desaki Y. Ichimura K. Shirasu K.. (2007). CERK1, a LysM receptor kinase, is essential for chitin elicitor signaling in *Arabidopsis*. Proc. Natl. Acad. Sci. U.S.A. 104, 19613–19618. 10.1073/pnas.0705147104 18042724 PMC2148337

[B27] Petutschnig E. K. Jones A. M. Serazetdinova L. Lipka U. Lipka V. (2010). The lysin motif receptor-like kinase (LysM-RLK) CERK1 is a major chitin-binding protein in arabidopsis thaliana and subject to chitin-induced phosphorylation. J. Biol. Chem. 285, 28902–28911. 10.1074/jbc.M110.116657 20610395 PMC2937917

[B28] Rinaudo M. (2006). Chitin and chitosan: properties and applications. Prog. Polym. Sci. 31, 603–632. 10.1016/j.progpolymsci.2006.06.001

[B29] Sánchez-Vallet A. Mesters J. R. Thomma B. P. H. J. (2014). The battle for chitin recognition in plant-microbe interactions. Fems Microbe. Rev. 39, 171–183. 10.1093/femsre/fuu003 25725011

[B30] Shibuya N. Ebisu N. Kamada Y. Kaku H. Cohn J. Ito Y. (1996). Localization and binding characteristics of a high-affinity binding site for N-acetylchitooligosaccharide elicitor in the plasma membrane from suspension-cultured rice cells suggest a role as a receptor for the elicitor signal at the cell surface. Plant Cell Physiol. 37, 894–898. 10.1093/oxfordjournals.pcp.a029030

[B31] Shibuya N. Kaku H. Kuchitsu K. Maliarik M. J. (1993). Identification of a novel high-affinity binding-site for N-acetylchitooligosaccharide elicitor in the membrane-fraction from suspension-cultured rice cells. FEBS Lett. 329, 75–78. 8354412 10.1016/0014-5793(93)80197-3

[B32] Shibuya N. Minami E. (2001). Oligosaccharide signalling for defence responses in plant. Physiol. Mol. Plant Pathol. 59, 223–233. 10.1006/pmpp.2001.0364

[B33] Shinya T. Nakagawa T. Kaku H. Shibuya N. (2015). Chitin-mediated plant-fungal interactions: catching, hiding and handshaking. Curr. Opin. Plant Biol. 26, 64–71. 10.1016/j.pbi.2015.05.032 26116978

[B34] Shinya T. Yamaguchi K. Desaki Y. Yamada K. Narisawa T. Kobayashi Y.. (2014). Selective regulation of the chitin-induced defense response by the *Arabidopsis* receptor-like cytoplasmic kinase PBL27. Plant J. 79, 56–66. 10.1111/tpj.12535 24750441

[B35] Tanaka S. Ichikawa A. Yamada K. Tsuji G. Nishiuchi T. Mori M.. (2010). HvCEBiP, a gene homologous to rice chitin receptor CEBiP, contributes to basal resistance of barley to *Magnaporthe oryzae*. BMC Plant Biol. 10:288. 10.1186/1471-2229-10-288 21190588 PMC3020183

[B36] Tsigos I. Martinou A. Kafetzopolus D. Bouriotis V. (2000). Chitin deacetylases: new, versatile tools in biotechnology. Trends Biotechnol. 18, 305–312. 10.1016/S0167-7799(00)01462-1 10856926

[B37] Trouvelot S. Héloir M.-C. Poinssot B. Gauthier A. Paris F. Guillier C.. (2014). Carbohydrates in plant immunity and plant protection: roles and potential application as foliar sprays. Front. Plant Sci. 5:592. 10.3389/fpls.2014.00592 25408694 PMC4219568

[B38] Vander P. Vårum K. M. Domard A. El Gueddari N. E. Moerschbacher B. M. (1998). Comparison of the ability of partially N-acetylated chitosans and chitooligosaccharides to elicit resistance reactions in wheat leaves. Plant Physiol. 118, 1353–1359. 9847109 10.1104/pp.118.4.1353 PMC34751

[B39] Wan J. Zhang X.-C. Neece D. Ramonell K. M. Clough S. Kim S.-Y.. (2008). A LysM receptor-like kinase plays a critical role in chitin signaling and fungal resistance in *Arabidopsis*. Plant Cell 20, 471–481. 10.1105/tpc.107.056754 18263776 PMC2276435

[B40] Yin H. Du Y. (2009). A new method for identification the oligochitosan binding protein on tobacco plasma membrane. Comp. Biochem. Physiol. A 153, S217 10.1016/j.cbpa.2009.04.606

[B41] Yin H. Du Y. Zhang J. (2009). Low molecular weight and oligomeric chitosans and their bioactivities. Curr. Top. Med. Chem. 9, 1546–1559. 10.2174/156802609789909795 19903163

[B42] Yin H. Li Y. Zhang H.-Y. Wang W.-X. Lu H. Grevsen K. (2013). Chitosan oligosaccharides-triggered innate immunity contributes to oilseed rape resistance against *Sclerotinia Sclerotiorum*. Int. J. Plant Sci. 174, 722–732. 10.1086/669721

[B43] Yin H. Zhao X. Du Y. (2010). Oligochitosan: a plant diseases vaccine-A review. Carbohydr. Polym. 82, 1–8. 10.1016/j.carbpol.2010.03.066

[B44] Zeng L. Velásquez A. C. Munkvold K. R. Zhang J. Martin G. B. (2012). A tomato LysM receptor-like kinase promotes immunity and its kinase activity is inhibited by AvrPtoB. Plant J. 69, 92–103. 10.1111/j.1365-313X.2011.04773.x 21880077 PMC3240704

